# Supplementary figures and images for: Allele-specific silencing of a dominant SETX mutation in familial amyotrophic lateral sclerosis type 4
Source: bioRxiv. 2024 Oct 12:2024.10.11.617871. Preprint. [Version 1] doi: 10.1101/2024.10.11.617871 (PMC11483056; doi:10.1101/2024.10.11.617871)

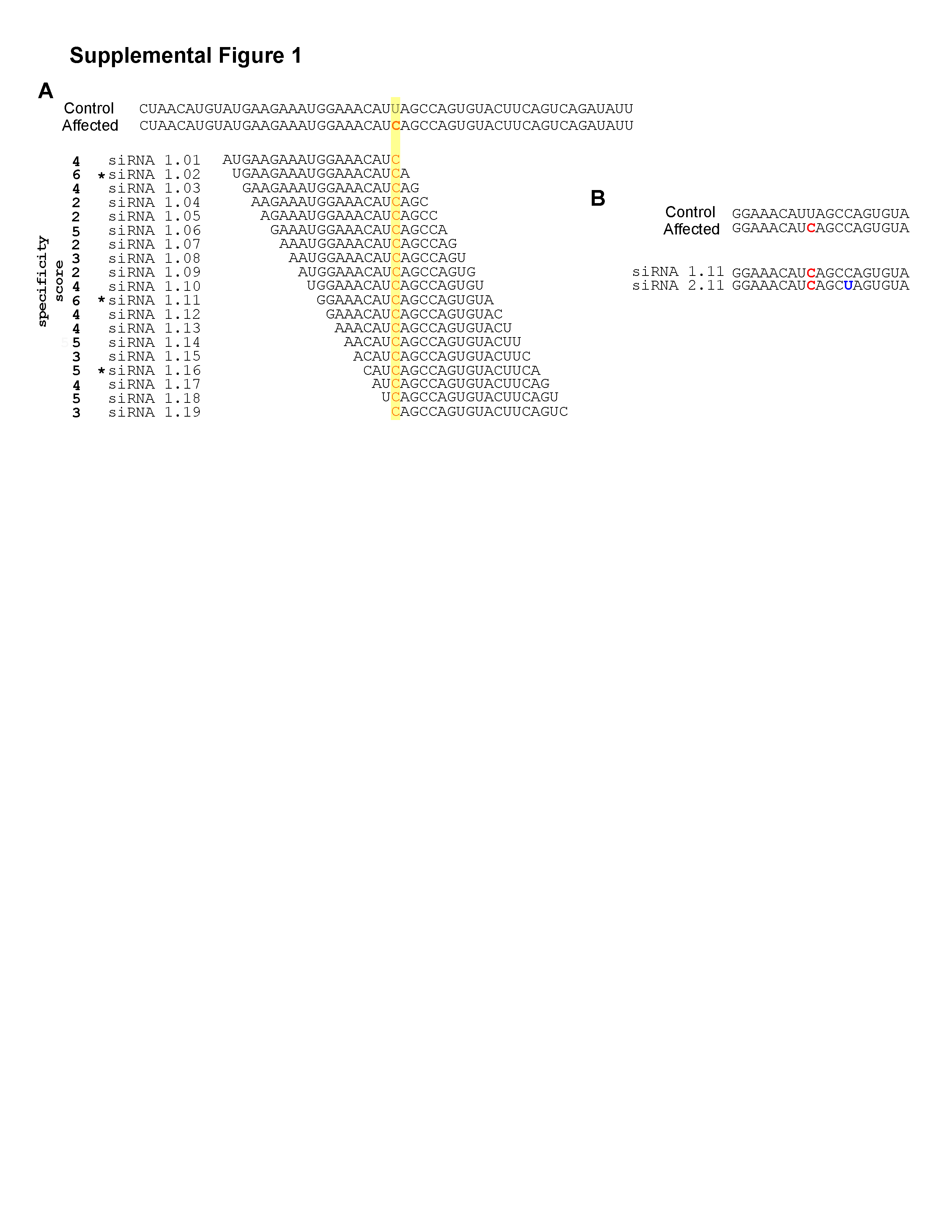

Supplement: Supplement 1 — (A) Control and ALS4 sequence at coding sequence position 1166 highlighted in yellow with the mutant allele variant shown in red. siRNAs 1.02, 1.11, and 1.16 had high specificity scores and were chosen for further analysis. (B) siRNA 2.11 was generated with an additional base mismatch shown in blue to assist in allele specificity. [file media-1.tif]

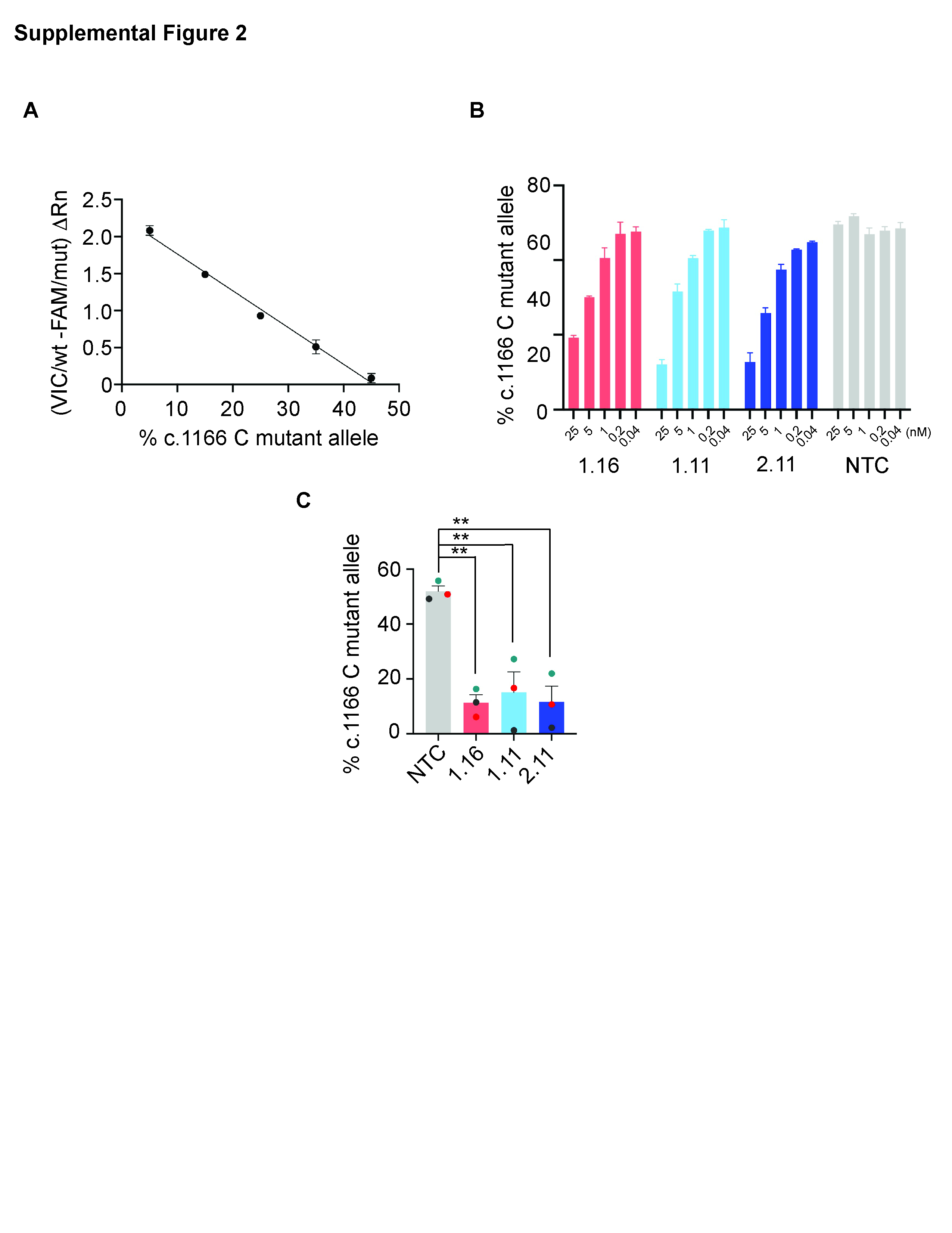

Supplement: Supplement 2 — (A) Representative standard curve using the SETX c.1166 T>C SNP genotyping probe with a dilution series of the ALS4 and control fibroblast cDNA. (B) Dose-dependent knockdown of the mutant allele of L389S in an ALS4 patient fibroblast line in fibroblasts treated for 72 hrs with increasing doses of siRNA. (C) Quantification of knockdown of the mutant L389S SETX allele in three patient fibroblast lines (each indicated with a separate color circle) using 25 nM of siRNA. [file media-2.tif]

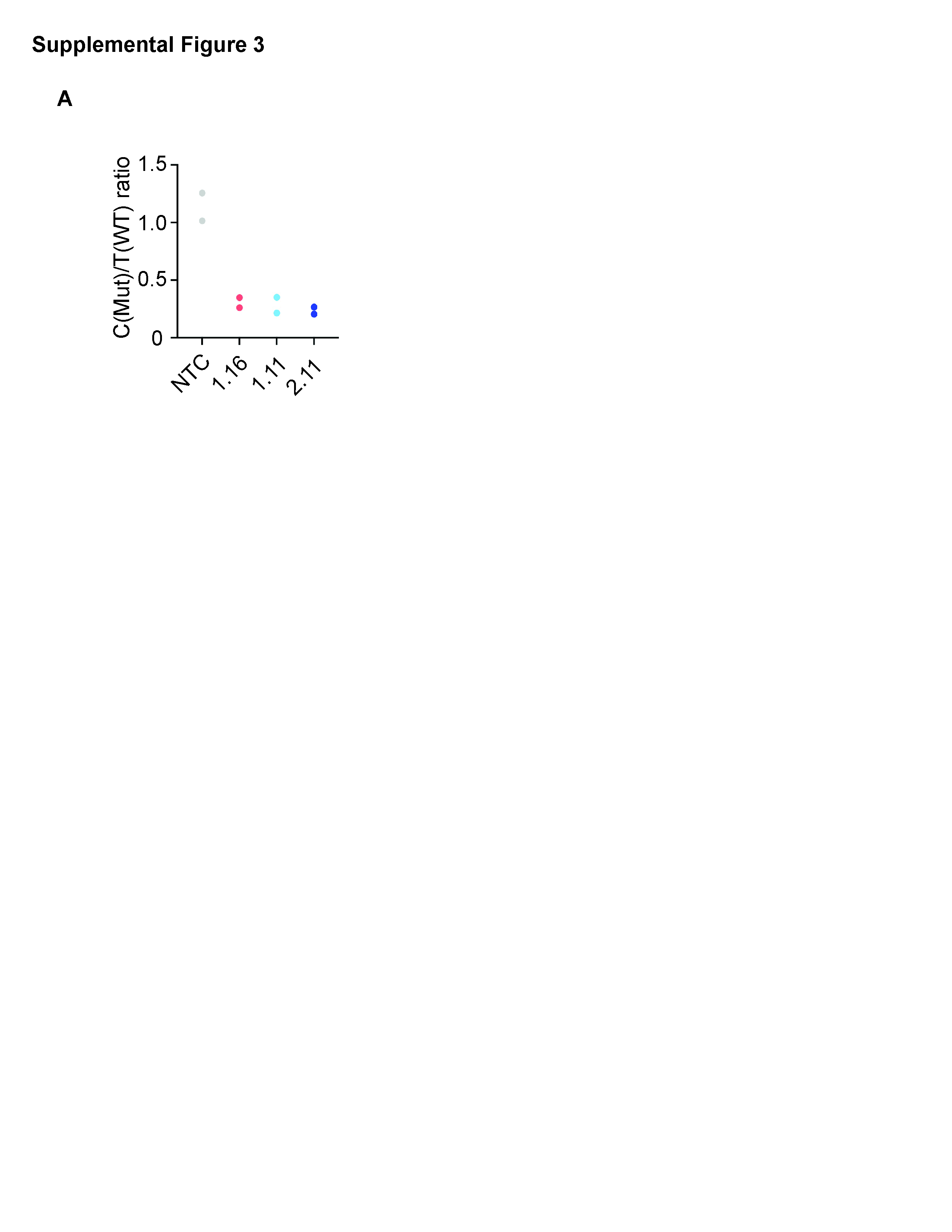

Supplement: Supplement 3 — (A) Quantification of Sanger sequencing of cDNA from treated fibroblasts showing the relative abundance of the mutant allele (C-bearing) to the wild-type allele (T-bearing). Each circle represents an independent replicate. [file media-3.tif]
